# Low-dose tissue plasminogen activator in the treatment of a massive pulmonary thromboembolism in a colon cancer patient treated with bevacizumab: A case report

**DOI:** 10.3892/ol.2014.2568

**Published:** 2014-09-26

**Authors:** FATMA SEN, YUSUF KARAVELIOGLU, ARIF ARISOY

**Affiliations:** 1 Department of Oncology, Hitit University, Corum Educational and Research Hospital, Corum 19100, Turkey; 2 Department of Cardiology, Hitit University, Corum Educational and Research Hospital, Corum 19100, Turkey

**Keywords:** pulmonary thromboembolism, bevacizumab, tissue plasminogen activator

## Abstract

The current study describes the fibrinolytic treatment of a patient exhibiting an acute massive pulmonary thromboembolism, who was also receiving a bevacizumab-based combination regimen for metastatic colon cancer. The administration of bevacizumab has been associated with an increased risk of venous thromboembolic events and bleeding in cancer patients. However, there is insufficient data regarding the safety and activity of thrombolytic agents in cancer patients receiving bevacizumab-based therapy. In the present case, despite the increased risk of bleeding, low-dose and prolonged tissue plasminogen activator infusion was effectively and reliably applied to treat a massive pulmonary embolism, which resulted in hemodynamic instability in the patient.

## Introduction

Cancer often leads to hypercoagulability, which may result in a variety of clinical manifestations, including migratory superficial thrombophlebitis, venous thrombosis, non-bacterial thrombotic endocarditis and disseminated intravascular coagulation. The release or expression of procoagulants by tumor cells and the demonstration of procoagulant activity by monocytes, platelets and endothelial cells are hypothesized to cause vascular complications in patients exhibiting malignancies. The common treatments for cancer include surgery, high-dose chemotherapy, bone marrow transplantation and numerous chemotherapeutic regimens. In addition, an indwelling central venous catheter may be used, which may significantly increase the risk of thrombotic events in patients with malignancy (1).

Angiogenesis inhibitors are increasingly administered for the treatment of patients with malignancies. Bevacizumab is a humanized monoclonal antibody that inhibits angiogenesis by inhibiting vascular endothelial growth factor (VEGF)-A. Bevacizumab was initially approved in 2004 for combinational use with standard chemotherapy for metastatic colon cancer treatment. However, the use of bevacizumab has been associated with an increased risk of venous thromboembolic events and bleeding in cancer patients (2,3). Currently, there is insufficient data regarding the safety and activity of thrombolytic agents in the treatment of massive pulmonary thromboembolisms that have developed in cancer patients undergoing bevacizumab-based therapy.

In the present study, the case of a patient with an acute massive pulmonary thromboembolism is presented, who received fibrinolytic treatment, whilst receiving a bevacizumab-based combination regimen for metastatic colon cancer. Written informed consent was obtained from the patient.

## Case report

**Figure 1:**
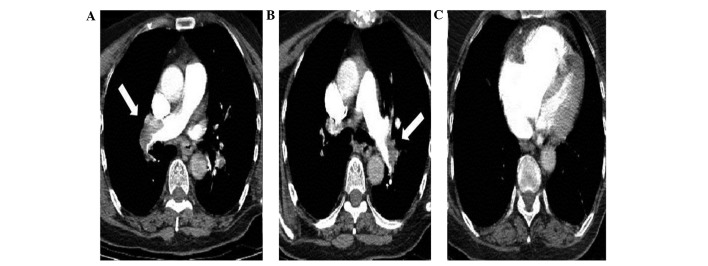
Pulmonary thromboembolism on computed tomography scan. (A) Right middle and lower pulmonary artery branch emboli. (B) Left pulmonary artery emboli. (C) Right ventricular dilatation (right ventricular cavity wider than left ventricular cavity in short axis).

**Figure 2:**
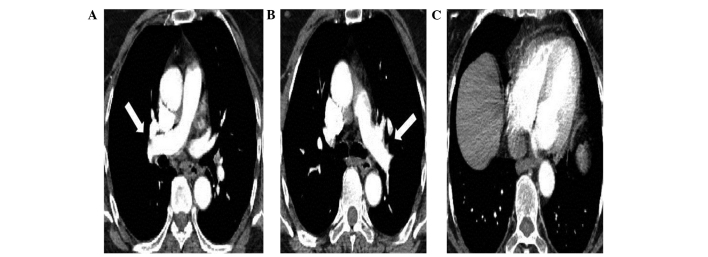
Computed tomography scan following thrombolytic therapy. (A) Reperfusion of the branches of the (A) right and (B) left pulmonary arteries. (C) Normalization of the diameter of the left and right ventricular cavities.

In September 2013, a 66-year-old female with a metastatic colon carcinoma was admitted to the emergency department of Hitit University, Corum Educational and Research Hospital (Corum, Turkey) with acute dyspnea, palpitations and dizziness. The patient exhibited hypertension, however, the patient’s medical history did not include smoking, diabetes mellitus, ischemic heart disease or any thrombotic disease. The patient underwent nine cycles of the FOLFIRI (90 min intravenous infusion of 180 mg/m^2^ irinotecan, 400 mg/m^2^ fluorouracil and 400 mg/m^2^ leucovorin, followed by a 46 h intravenous infusion of 2,400 mg/m^2^, entire regimen delivered twice a week, for 18 weeks) plus bevacizumab combination therapy. The patient’s symptoms developed 10 days following the last cycle of chemotherapy. On physical examination the patient’s blood pressure was 70/50 mmHg and heart rate was 120 bpm. The patient exhibited tachypnea, tachycardia, jugular venous distention and a systolic 2/6 murmur was identified on all cardiac points. An emergency two-dimensional ultrasonographic echocardiography revealed right heart dilatation, moderate tricuspid regurgitation and pulmonary hypertension. Thus, as the patient was considered to have a high risk of pulmonary embolism [PE; Wells score (4), 7 points], a PE was suspected. Thoracic computed tomography (CT) angiography demonstrated a bilateral pulmonary arterial embolism (Fig. 1). The patient was diagnosed with a massive PE and hemodynamic instability. Due to the patient’s malignancy and risk of bleeding, prolonged low-dose thrombolytic therapy [25 mg tissue plasminogen activator (tPA) infusion for 6 h] was administered to the peripheral vein, according to a previous study by Aykan *et al* (5), rather than a standard thrombolytic regime. Following treatment, there was an increase in blood pressure (100/70 mmHg) and improvement in the patient’s clinical condition. No bleeding complications were observed. Control echocardiography revealed an improvement in the right heart chambers and a decrease in pulmonary pressure. The CT angiography revealed that the peripheral vascular bed was reperfused (Fig. 2). Additionally, Doppler ultrasound revealed acute deep vein thrombosis (DVT) in the right lower extremities, which was considered to be the source of the pulmonary embolism.

## Discussion

Numerous studies have indicated that progression-free survival and overall survival are improved by the administration of bevacizumab, in combination with various chemotherapy regimens, for patients with metastatic colon cancer (5,6). The class-effects of VEGF axis inhibition include cardiovascular effects, for example hypertension and left ventricular dysfunction, and non-cardiovascular effects, including proteinuria, delayed wound healing, gastrointestinal perforation, fatigue and dysphonia. In addition, the administration of bevacizumab in conjunction with chemotherapy is associated with an increased risk of thromboembolic and bleeding events (2,3). PE occurs in 2–5% of cases where bevacizumab and chemotherapy are used in combination (6,7). In the present study, the patient was diagnosed with a massive PE and DVT following bevacizumab combination therapy.

It is recommended that thrombolytic therapy is followed by anticoagulation therapy, rather than anticoagulation alone, for patients exhibiting acute PE who are persistently hypotensive as a result of the PE (systolic blood pressure <90 mmHg or a decrease in systolic blood pressure of ≥40 mmHg from baseline) and who do not exhibit an increased risk of bleeding. The results of previous studies indicate that thrombolytic therapy leads to early hemodynamic improvement, however, is associated with an increased risk of major bleeding (8,9).

Results regarding the effect of thrombolytic therapy on the improvement of mortality are controversial. Despite the inconsistent results of controlled clinical trials, one observational study of 72,230 unstable patients with acute PE revealed that thrombolytic therapy was associated with lower all-cause mortality when compared with no treatment (15 vs. 47%, respectively) and lower mortality attributable to PE when compared with no treatment (8.4 vs. 42%) (10). However, observational results obtained from the same population revealed that thrombolytic therapy was underutilized and less likely to be administered in older patients (aged >60 years) and in patients with comorbid conditions, highlighting a possible lack of confidence exhibited by clinicians regarding the use of thrombolytic therapy (11). While the reported effect size is large in the two above-mentioned studies, the observational design and the potential influence of bias demonstrates that the efficacy of thrombolytic therapy in this clinical setting remains unclear. Although bevacizumab may increase the risk of bleeding, due to the patient’s hemodynamic instability in the present case, thrombolytic therapy was initiated immediately.

In patients exhibiting massive pulmonary embolisms, the guidelines for conducting thrombolytic therapy recommend peripheral venous administration of 100 mg tPA for 2 h (12). The risk of bleeding, a significant complication of thrombolytic therapy, has been reported to be as high as 20% in older patients with a large body mass index and a history of previous catheterization (13–15). However, to the best of our knowledge, no sufficient data exists regarding a prolonged low-dose tPA regime. In previous studies, it has been demonstrated that prolonged low-dose tPA may be effectively and reliably administered to elderly patients in whom the risk of bleeding is high (16), in patients with a prosthetic valve (17) or in patients who exhibit hemoptysis secondary to an embolism (18) without an increased risk of bleeding.

In conclusion, thrombolytic therapy in patients with malignancies is associated with an increased risk of bleeding, regardless of the presence of metastasis. Certain chemotherapeutic agents, including bevacizumab, may elevate the risk of bleeding. However, in the present study, despite the increased risk of bleeding, low-dose and prolonged tPA infusion was effectively and reliably administered in a patient with a massive PE, although it did result in hemodynamic instability.
